# Supplementary material for: Personal and perceived stigma towards mental disorders among attendants of patients with mental illness in selected health facilities of Bangladesh
Source: PLoS One. 2025 Oct 17;20(10):e0333959. doi: 10.1371/journal.pone.0333959 (PMC12533838; doi:10.1371/journal.pone.0333959)
Supplement: S1 Table — (PDF) [file pone.0333959.s003.pdf]

S1 Table. Multivariate analysis of this study

Variable	Positively scored domains					Negatively scored domains				
	Df	Sum Sq	Mean Sq	F value	P value	Df	Sum Sq	Mean Sq	F value	P value
Domain	3	361	120.5	0.58	0.63	2	0.0	0.0	0.0	1.0
Statements	16	1358	84.9	0.41	0.98	4	0.0	0.0	0.0	1.0
Residuals	120	25070	208.92			42	13701	326.2		
