# Supplementary material for: Personal and perceived stigma towards mental disorders among attendants of patients with mental illness in selected health facilities of Bangladesh
Source: PLoS One. 2025 Oct 17;20(10):e0333959. doi: 10.1371/journal.pone.0333959 (PMC12533838; doi:10.1371/journal.pone.0333959)
Supplement: Attachment — Submitted filename: Resonse to Reviewers.docx [file pone.0333959.s005.docx]

To

The Editors

PLOS ONE

Dear Editors

Thank you for sending the comments on our manuscript titled “Personal and perceived stigma towards mental disorders among attendants of patients with mental illness in selected health facilities of Bangladesh”. On behalf of my team, I would also like to thank you and the reviewers for your insightful comments, which will help us improve our article. Please locate the revised drafts and the comments' corresponding responses in the following page.

Thank you.

Your Sincerely

Labida Islam

| **Academic Editor** |  |
| --- | --- |
| 1. Please ensure that your manuscript meets PLOS ONE's style requirements, including those for file naming. | Thank you for your feedback. Necessary revision was made according to the PLOS ONE’s requirements. |
| 2. Thank you for stating the following financial disclosure:  The study was funded by the Directorate General of Health Services (DGHS) Bangladesh (invitation ref no: DGHS/LD/NCDC/Procurement plan/RPA (GOB) Service/2018-19/ 2018/5217/SP-05) under the name of Saidur Rahman Mashreky as Principal Investigator. Please state what role the funders took in the study. If the funders had no role, please state: "The funders had no role in study design, data collection and analysis, decision to publish, or preparation of the manuscript." If this statement is not correct you must amend it as needed.  Please include this amended Role of Funder statement in your cover letter; we will change the online submission form on your behalf. | Thank you once again. The funders’ role was stated in the cover letter according to your suggestion. |
| 3. Please amend your list of authors on the manuscript to ensure that each author is linked to an affiliation. Authors’ affiliations should reflect the institution where the work was done (if authors moved subsequently, you can also list the new affiliation stating “current affiliation:….” as necessary).’ | Thank you. Author list was amended where the work was done and according to their current affiliation in the line number 8 to 18. |
| 4. Please remove all personal information, ensure that the data shared are in accordance with participant consent, and re-upload a fully anonymized data set. | Thank you once again. After removing all personal information re-upload was done. |
| 5. We are unable to open your Supporting Information file Data set.sav. Please kindly revise as necessary and re-upload. | Thank you once again. Necessary revision was made and re-upload was done. |

| **Reviewer 1** | |
| --- | --- |
| 1. What are the mental illnesses of the patients in this study? Please also provide the characteristics of the subject patients. | Thank you for your comment. We did not explore the types of mental illness and include this in the limitation section in the line number 357.  According to your suggestion characteristics of the subject patients was included in the table 1 in the line number 168 to 175. |
| 2. Please provide details about the relationship between the attendant and the patient. (e.g., depth of relationship, duration of association, etc.) | Thank you once again. Depth of relationship was stated in the table 1 in the line number 173 to 174. |

| **Reviewer 2** | |
| --- | --- |
| The manuscript, “Personal and perceived stigma towards mental disorders among attendants of patients with mental illness in selected health facilities of Bangladesh,” addresses an important and underexplored aspect of mental health in low-resource settings. The topic is of considerable public health relevance, particularly in low- and middle-income countries like Bangladesh, where access to mental health care remains limited and stigma poses a major barrier to treatment. The mixed-methods design is appropriate, capturing both quantitative (n=176) and qualitative (n=20) perspectives. The use of the Day’s Mental Illness Stigma Scale, coupled with in-depth interviews, demonstrates an effort to understand nuanced perceptions across seven domains. Notably, the study highlights high levels of stigma in domains such as hygiene, relationships, and anxiety, while more positive attitudes were observed regarding treatability and trust in professional care—findings that could inform culturally sensitive interventions and awareness efforts. |  |
| Despite its strengths, the manuscript has several methodological and analytical limitations that should be addressed before it can be considered for publication. The use of convenience sampling, without adequate justification, introduces significant selection bias and limits the study’s generalizability. Additionally, the absence of stratification by key sociodemographic factors (e.g., urban/rural location, socioeconomic status, or diagnostic category) weakens the conceptualization of the study setting and undermines the representativeness of the findings. The rationale for the sample sizes in both components is not provided, and while the authors mention achieving thematic saturation in the qualitative data, no supporting documentation (e.g., saturation matrix or coding framework) is presented. Moreover, it is unclear whether the scale was validated for the local context. Providing details on translation, cultural adaptation, and psychometric validation—or citing appropriate sources—would strengthen the manuscript’s measurement validity. | - Thank you for your valuable comment. We selected ten tertiary level health facilities of Bangladesh among them two of the health facilities were specialized mental health care centres. These ten health facilities were all tertiary-level mental health centres in Bangladesh, where patients from across the country receive care. However, data from community settings may differ from facility data. Thus, we can say it is a national representative study; however, the study was less generalizable as data from community settings and in health facility settings are differ from each other. Thus, we include this in our study limitation settings in the line number 356 to 363. - The justification for convenient sampling given in the method section in the line number 92 to 96 and in the limitation section in the line number 356 to 363. - Back in 2019, as people are not that much aware about mental illness and we collected data from 2^nd^ March 2019 to 20^th^ April 2019, so during this time frame whatever patient we found with mental illness we included in this study considering their willing participation and the revision was made in the line number 87 to 88 and in the line number 99 to 100 - Qualitative data file was provided as a supplementary document in the line number 509. - The scale was validated for the local context and necessary revision was made in the data collection instrument section in the line number 103 to 106. |
| The data analysis also requires enhancement. The manuscript presents only descriptive statistics, with no mention of inferential testing (e.g., p-values, confidence intervals) or specification of the statistical tests used to compare subgroups. The absence of multivariate analysis limits understanding of potential confounders, especially regarding the influence of age, sex, and education on stigma scores.  On the qualitative side, while the thematic findings are informative, the analysis lacks detail. The coding strategy, analytical framework, and measures to ensure reliability (such as double coding or inter-coder agreement) are not described. This limits confidence in the qualitative conclusions, which appear anecdotal rather than systematically derived. Furthermore, integration between qualitative and quantitative results is limited and should be improved to enrich interpretation. | - Thank you once again. Multivariate analysis was carried out as per your comment, we revised it in the line number 269 to 273 and include it in the supplementary document in the line number 510. - Necessary changes were made in the qualitative data analysis section in the line number 147 to 152: “*The in-depth interviews were transcribed verbatim and translated into English. The data was then summarized and analyzed manually by two researchers as per a guideline consisting of a list of a priori codes and themes, which were based on literature reviews. Any discrepancies or differences in opinions were discussed and resolved by the researchers. Thematic analysis was carried out in this study, which coincides with the quantitative results of this study. Relevant quotations were used to support the descriptive analysis of the data”.* |
| Several sections of the manuscript would benefit from editing for clarity, conciseness, and grammar. The discussion section often reiterates results without deeper engagement or comparison with existing literature. Some citations are outdated or drawn from non-peer-reviewed sources; incorporating recent, peer-reviewed research would improve the scientific grounding. Ethical considerations also require greater detail—particularly regarding informed consent procedures, participant anonymity, and emotional safeguards during interviews. While the limitations are acknowledged, the discussion does not adequately address the implications of convenience sampling, diagnostic ambiguity, or cross-sectional design. These factors constrain the study’s capacity to make causal inferences and should be discussed more explicitly. Overall, the paper talks about an important topic and provides helpful initial findings, but it needs major revisions—especially in explaining the study design, improving analysis, and being clearer in reporting—to meet the publication standards of PLOS ONE. I believe that with these significant changes and clearer reporting, this study could substantially add to the research on mental health stigma in low-resource settings. | - Thank you again for your valuable comment. As per your instructions discussion section was revised in the line number 282 to 355. - Citations were updated according to your comments and change was made in the reference section in the line number 379 to 505. - According to your suggestion ethical clearance part was revised as follows *“Written informed consent was obtained from each study participant. Participants had the right to withdraw from the study at any point of data collection. Attempts were made by the researchers to maintain scientific rigour, accuracy, impartiality, emotional safeguarding and anonymity where appropriate during data collection. Qualitative data was collected in a closed room to ensure the anonymity of the attendees”* in the line number 155 to 160. |
