# Supplementary material for: Personal and perceived stigma towards mental disorders among attendants of patients with mental illness in selected health facilities of Bangladesh
Source: PLoS One. 2025 Oct 17;20(10):e0333959. doi: 10.1371/journal.pone.0333959 (PMC12533838; doi:10.1371/journal.pone.0333959)
Supplement: Attachment — Submitted filename: Resonse_to_Reviewers_auresp_2.docx [file pone.0333959.s006.docx]

To

The Editors

PLOS ONE

Dear Editors

Thank you for sending the comments on our manuscript titled “Personal and perceived stigma towards mental disorders among attendants of patients with mental illness in selected health facilities of Bangladesh”. On behalf of my team, I would also like to thank you and the reviewers for your insightful comments, which will help us improve our article. Please locate the revised drafts and the comments' corresponding responses in the following page.

Thank you.

Your Sincerely

Labida Islam

| **Reviewer 2** | |
| --- | --- |
| Thank you for the revised submission. The authors have addressed some earlier concerns and made improvements to the manuscript. The introduction now presents a clearer rationale for investigating stigma in the Bangladeshi context, and the overall language has improved. These revisions show a good-faith effort to engage with reviewer feedback and are appreciated.  However, several key issues remain unresolved. Some of the claims made in the author response, such as the inclusion of multivariate analysis and the validation of the stigma scale, are not supported by the actual content of the manuscript. |  |
| 1. On lines 103 to 106, the authors mention that the Day’s Mental Illness Stigma Scale was translated into Bengali and back-translated into English by bilingual researchers. While this is an important step, the manuscript provides no evidence of psychometric validation in the Bangladeshi context. There is no mention of reliability statistics, such as Cronbach’s alpha, no description of how the scale was culturally adapted, and no citation of any prior validation study. Since the scale forms the core of the study’s quantitative component, this omission limits the reliability of the findings. | Thank you for your valuable comment. According to your suggestion necessary revision was made in the line number 106 to 109. Prior to data collection, a pretest of the questionnaire was conducted in the above-selected health facilities among 30 attendants. The scale was utilized in the study since there aren't any culturally validated measures that measure the stigma associated with mental illness in Bangladesh.  Study conducted in Bangladesh using the same scale showed that prior to the use in their study, the scale was administered to a sample of 50 participants (excluding the main sample) in both rural and urban areas to understand the comprehensibility of items. No item was found to be difficult to understand, therefore, no change was required. The Cronbach’s alpha for the total scale in the present study was 0.67. The Cronbach’s alphas for the subscales in the present study were 0.71, 0.50, 0.52, 0.76, 0.46, 0.52, and 0.74, respectively. They assessed test–retest reliability of the scale on a sample of an additional 100 participants in both settings with a gap of 2 weeks. The test–retest reliability coefficient for the present study was found to be r = 0.81 at p < 0.01. |
| 2. Reference 17, which the authors cite in support of the scale, appears to point to a general Google Scholar search rather than a valid source. If the intention was to cite the Bangladesh National Mental Health Survey, that reference needs to be correctly formatted and clearly explained. Even if the survey used the Day’s scale, its use alone does not imply validation. If validation was conducted, the authors should cite the appropriate report and describe the findings. Otherwise, they should consider conducting a basic psychometric analysis themselves or referencing another study, such as the one listed as reference 26 or similar publications that have used the scale in Bangladesh. The current citation does not provide adequate justification for the scale’s validity. | Thank you once again. Reference 17 was corrected and revised and another reference was put with 17 no reference which was previously 26 number but now it was 18 number in the line number 106 to 107.  The scale was used in National Mental Health survey but they only validated the tool by translation and back-translation. But in another study, they used Cronbach’s alpha test. prior to the use in their study, the scale was administered to a sample of 50 participants (excluding the main sample) in both rural and urban areas to understand the comprehensibility of items. No item was found to be difficult to understand, therefore, no change was required. The Cronbach’s alpha for the total scale in the present study was 0.67. The Cronbach’s alphas for the subscales in the present study were 0.71, 0.50, 0.52, 0.76, 0.46, 0.52, and 0.74, respectively. They assessed test–retest reliability of the scale on a sample of an additional 100 participants in both settings with a gap of 2 weeks. The test–retest reliability coefficient for the present study was found to be r = 0.81 at p < 0.01. |
| 3. In their response, the authors state that multivariate analysis was performed. However, the revised manuscript does not include any such analysis. The results section presents only descriptive statistics, including means, standard deviations, and frequencies. No regression models, p-values, confidence intervals, or adjusted comparisons are provided. This is a major gap, especially since the authors draw interpretive conclusions across demographic groups.  An ANOVA is included in the supplementary file (S1 Table), but this only compares mean scores across stigma domains within the scale. It does not examine how respondent characteristics, such as education or gender, influence stigma scores. This was the focus of the requested multivariate analysis and remains unaddressed. Without this level of analysis, it is not possible to understand the independent contributions of key variables or to control for confounding. | Thank you for your valuable comment. As per your suggestion multivariate analysis was carried out and we put it as table 5 in the line number 304 to 310. |
| 4. The methods section notes that SPSS was used, but the actual statistical procedures are not described. For example, Table 3 presents stigma scores by education level, but the manuscript does not report whether the differences are statistically significant. Statements which suggests that participants with higher education showed less stigma, are not supported by formal statistical testing. | Thank you. Necessary revision was made in the line number 304 to 310. |
| 5. The qualitative component also lacks sufficient methodological detail. While the manuscript states that thematic saturation was reached after 20 interviews, no explanation is provided for how this decision was made. There is no mention of how saturation was defined or assessed, and there is no coding framework, saturation matrix, or supporting evidence. This makes it difficult to evaluate the rigor of the analysis.  The authors also do not describe how codes were developed, how many researchers were involved in the coding process, or whether inter-coder reliability was assessed. There is no mention of using any established qualitative analysis framework. Without these details, it is hard to judge the trustworthiness of the qualitative findings. Additionally, the integration of qualitative and quantitative results is minimal. The findings are presented separately rather than used to build a coherent narrative that draws on both data types. | - Thank you once again. As qualitative data collecting was no longer generating new themes, insights or any codes relevant to our research question and the redundancy was consistent, with the judgement of lead researcher the qualitative study team decided to stop data collection after 40 interviews. This thematic saturation aligns with recommendations from Guest et al. that data saturation can often be achieved within 12-15 interviews for relative similar participant groups. The necessary revision was made in the line number 120 to 124. Supporting evidence was given in the supplementary document S2 File in the line number 540. - To enhance reliability, two researchers independently coded four of the same initial transcripts, and the results were compared for the establishment of the codes in the line number 159 to 161. - Codes were developed starting with a broad and descriptive familiarization. Then they were refined into meaningful categories and then grouped into themes in the line number 157 to 159. After that, a documentation was made with a codebook, which has been provided in the supplementary document. - Inter-coder agreement was assessed periodically by the joint coding an additional transcript every 5 interviews. (Cohen’s kappa calculation to monitor consistency where k= 0.82 which indicates substantial agreement in the line number 162 to 163. - Qualitative data analysis followed Braun and Clarke six phase framework for thematic analysis and revision was made in the line number 162 to 163. - Integration was done according to your suggestion in the line number 227 to 229, 240, 258 to 259 and 279. |
| 6. The sampling approach also remains a concern. The study uses convenience sampling from three health facilities. While this is acknowledged briefly, its implications are not discussed in sufficient depth. There is no reflection on how this may affect representativeness or introduce bias. The authors also do not stratify their analysis by meaningful variables, such as type of mental illness, rural versus urban setting, or the relationship between the attendant and the patient. These factors could provide important context for interpreting stigma but are not explored. | Thank you for your comment. We selected ten tertiary level health facilities of Bangladesh among them two of the health facilities were specialized mental health care centres, while the rest of eight medical college hospitals were selected based on the availability of mental health services. These Ten health facilities were all tertiary-level mental health centres in Bangladesh, where patients from across the country receive care in the line number 88 to 92.  In Bangladesh these ten tertiary facilities are the only facilities who treat patients with mental illness and as we mentioned before the two facilities are the only specialized mental health institutes. In this study we included all of them thus we can say it a representative sample.  The relationship between the attendant and the patient was discussed in the table 1 in the line number 187 to 188. Though we only explore this relationship for the qualitative part.  Type of mental illness and urban vs rural was not explored in this study and we include this limitation in the limitation part in the line number 386 to 388. |
| 7. Lastly, some language in the manuscript still implies causation, which is inappropriate for a cross-sectional study. Since the data are observational, the conclusions should be limited to associations. | Thank you once again for your valuable comment. Revision was made according to your suggestion in the line number 395 to 399. |
